# Deciphering the role of rapamycin in modulating decidual senescence: implications for decidual remodeling and implantation failure

**DOI:** 10.1007/s10815-024-03207-5

**Published:** 2024-07-27

**Authors:** Remziye Kendirci-Katirci, Leyla Sati, Ciler Celik-Ozenci

**Affiliations:** 1 Department of Histology and Embryology, School of Medicine, Akdeniz University, Antalya, Turkey; 2 Department of Histology and Embryology, School of Medicine, Koc University, Istanbul, Turkey; 3 Koc University Research Center for Translational Medicine (KUTTAM), Istanbul, Turkey

**Keywords:** Human decidualization, Decidual senescence, Rapamycin, Implantation, DIO2

## Abstract

**Purpose:**

Physiological decidual senescence promotes embryo implantation, whereas pathological decidual senescence causes many pregnancy pathologies. The aim of this study was to evaluate the effect of rapamycin on decidual cell subpopulations and endometrial function in physiological and induced senescence and to investigate the decidual cell subpopulations present in physiological conditions during early pregnancy and implantation in mice.

**Methods:**

Control, physiological decidualization (0.5 mM cAMP and 1 μM MPA added), and induced senescence (0.1 mM HU added) models with and without 200 nM rapamycin treatment were established using a human endometrial stromal cell line, and decidual cell subpopulations were analyzed by immunofluorescence and flow cytometry. The human extravillous trophoblast cell line AC-1M88 was also cultured in decidualization models, and spheroid expansion analysis was performed. In in vivo studies, decidual cell subpopulations were analyzed by immunofluorescence during early mouse pregnancy.

**Results:**

The results revealed that rapamycin decreased DIO2 and β-GAL expressions in physiological and induced senescence without FOXO1. Notably, in induced senescence, increased fragmentation was observed in AC-1M88 cells, and rapamycin treatment successfully attenuated the fragmentation of spheroids. We showed that the FOXO1-DIO2 signaling axis can trigger decidual senescence during early gestation and days of implantation in mice.

**Conclusions:**

Our study underlines the importance of rapamycin in modulating decidual cell subpopulations and endometrial tissue function during decidual senescence. The information obtained may provide insight into the pathologies of pregnancy seen due to decidual senescence and guide better treatment strategies for reproductive problems.

**Supplementary Information:**

The online version contains supplementary material available at 10.1007/s10815-024-03207-5.

## Introduction

Acute (physiological) cellular senescence is an evolutionarily conserved arrest of cell growth with roles in wound healing, tissue remodeling, and embryonic development [1]. During acute cellular senescence, acutely senescent cells arise and produce a transient senescence-associated secretory phenotype (SASP) that includes pro-inflammatory factors, cytokines and chemokines, growth factors, and extracellular matrix (ECM) proteins. Acutely senescent cells are cleared by immune cells, especially natural killer (NK) cells [2–5].

Recent studies highlight the importance of physiological decidual senescence resulting from replication stress in endometrial stromal cells during decidualization and the acute senescent decidual cells that arise during this process [6, 7]. Both in vitro and in vivo studies reveal the formation of senescent decidual cells during decidualization due to FOXO1 activation via PKA and progesterone pathways [3, 8]. Senescent decidual cells, which morphologically resemble decidual cells, are stress-intolerant, progesterone-resistant, and SASP-producing cells [7, 9]. Senescent decidual cells support the pro-inflammatory environment during decidualization, subsequently assist endometrial receptivity and embryo implantation in its continuation, and are subsequently cleared by recruited uterine natural killer (uNK) cells [6, 8].

In the process of chronic (pathological) cellular senescence, chronic senescent cells arise that contribute to age-related disorders [2]. In the process of decidualization, an overabundance of these cells in the environment causes pathological decidual senescence, leading to decidualization defects, prolonged pro-inflammatory phases, failure to support the anti-inflammatory phase, implantation failures, and many pregnancy and placenta-related pathologies, including recurrent pregnancy loss (RPL) [4, 10–12]. DIO2, which encodes iodothyronine deiodinase 2, has been recognized as a pivotal gene involved in the pathway leading to senescence [7]. Increased expression of DIO2, a marker of senescent decidual cells, has been detected in endometrial tissue and placenta samples of women with RPL [4]. Senescent cells exhibit elevated levels of β-GAL, which is encoded by GLB1, depending on the extent of cellular stress [13]. Increased levels of β-GAL in the decidual senescence may disrupt decidualization and create an unsuitable environment for embryo development, leading to pregnancy pathologies [7].

Hydroxyurea (HU) is a chemical compound used to induce cellular senescence. HU, which induces senescence in various cells in vitro, also leads to cellular senescence by suppressing ribonucleotide reductase activity, which is essential for successful decidual proliferation and healthy embryo implantation [14–16].

In senotherapy, which aims to reverse or stop the pathological state, SASP secretion is inhibited by senomorphic treatments [17]. Rapamycin, a specific inhibitor of mTOR signaling, has been shown to prolong life span in yeast, invertebrates, and mammals and, in vitro, to pharmacologically suppress cellular senescence [18–22].

As physiological decidual senescence plays a pivotal role in regulating endometrial fate during implantation, there is growing interest in innovative treatments for reproductive failure. However, the impact of rapamycin treatment on HU-treated induced senescence remains unknown. Thus, this study aims to explore the mechanisms of physiological and induced senescence, assess the effects of rapamycin on decidual cell subpopulations and endometrial functions, and examine decidual cell subpopulations during early pregnancy and implantation in mice under physiological conditions.

## Materials and methods

**Fig. 1:**
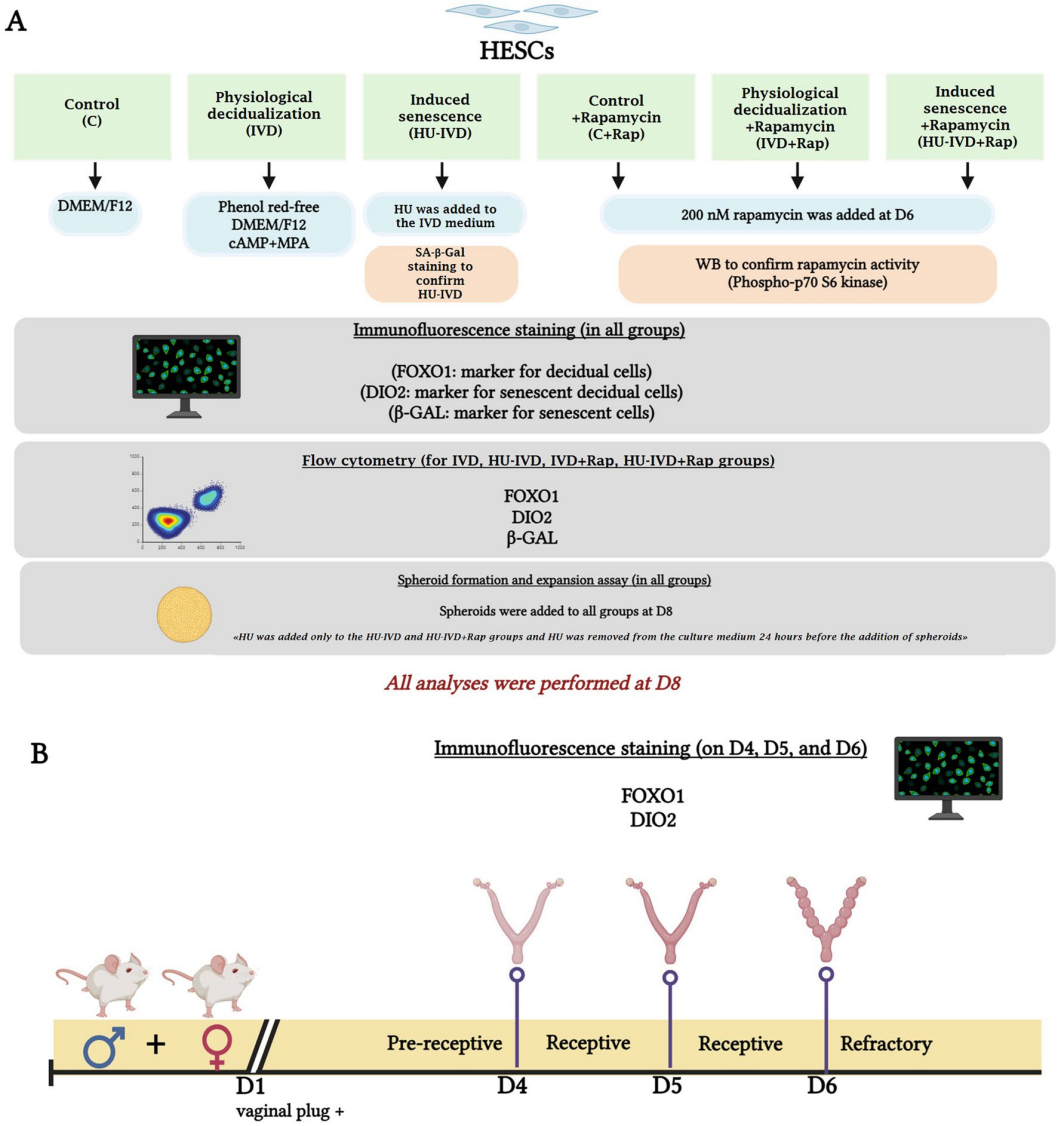
The experimental design. To evaluate the effect of rapamycin on decidual cell subpopulations and endometrial function during decidual senescence, in vitro physiological and HU-treated induced senescence models were established. Six groups were formed using human endometrial stromal cells (HESCs), including a control group (C), a group undergoing physiological decidualization (in vitro decidualization-IVD), a group undergoing induced senescence (HU-IVD), a control group treated with rapamycin (C + Rap), a group undergoing physiological decidualization treated with rapamycin (IVD + Rap), and a group undergoing induced senescence treated with rapamycin (HU-IVD + Rap) (**A**). Furthermore, in vivo studies were conducted in mice to identify decidual cell subpopulations that arise during early pregnancy and implantation under physiological conditions on different days of development (D4, D5, and D6) (**B)**. The illustration was generated using the BioRender software

Figure 1 summarizes our experimental design for both in vitro and in vivo experiments.

### Human endometrial stromal cell culture

Telomerase-immortalized HESCs were purchased from ABM (T0533) and cultivated as instructed by the supplier. HESCs were cultured in DMEM/F12 (Gibco, #11320033, USA) supplemented with 10% fetal bovine serum (FBS) (Gibco, #26140079, USA), 1% penicillin–streptomycin (Gibco, #15070063, USA), and 1% L-glutamine (Gibco, #25030081, USA) at 37 °C in a humidified incubator under 5% CO_2_. When the cells reached 80–90% confluency, they were passaged according to the guidelines provided by Applied Biological Materials as outlined in the study by Holdsworth-Carson and colleagues [23].

### *In vitro *decidualization

HESCs were first grown to 80% confluency to induce decidualization, and the day of this growth stage was designated as day 0 (D0) for all groups. For the physiological decidualization experiments, confluent monolayers of HESCs were cultured in phenol red–free DMEM/F12 (Thermo, #21041025, USA) supplemented with 2% DCC-FBS (Capricorn, #FBS-CS-12A, USA), 1% penicillin–streptomycin (Gibco, #15070063, USA), and 1% L-glutamine (Gibco, #25030081, USA), along with 0.5 mM 8-Bromo-cAMP (a stable, cell-permeable analogue of cAMP; Sigma Aldrich, #B5386, UK) and 1 μM medroxyprogesterone acetate (MPA), a synthetic progesterone derivative (Sigma Aldrich, UK), to induce a differentiated phenotype [7, 8]. The culture medium supplemented with 0.5 mM cAMP and 1 mM MPA was replaced every 48 h. In vitro decidualization was continued for 8 days to support the formation of mature decidual cells [7, 8]. The cell morphology was observed before and after decidualization. Morphological changes in the cells were monitored daily using phase contrast optics, and images were captured using an Olympus IX71 (Zeiss, Germany) inverted microscope.

### Induction of cellular senescence by HU

To prepare the induced senescence group, HU (Sigma Aldrich, #H8627, USA) was dissolved in purified water and added to a physiological decidualization medium consisting of phenol red-free DMEM/F12 with 2% DCC-FBS, 1% penicillin–streptomycin, and 1% L-glutamine, along with 0.5 mM 8-bromo-cAMP and 1 mM medroxyprogesterone acetate (cAMP + MPA). The culture medium was changed every 48 h. For optimization experiments, the induced senescence group was performed at three different concentrations of HU (0.1 mM, 0.2 mM, and 0.5 mM) [24] (Supplementary Fig. S1).

### Validation of HU treatment efficacy

An increase in the expression of lysosomal β-galactosidase is an indicator of the presence of senescent cells [13]. Therefore, we used CellEvent™ Senescence Green Staining (Invitrogen, #C10840, USA) under a fluorescence microscope following the manufacturer’s instructions for senescence associated-β-galactosidase (SA-β-Gal) expression as previously shown in the literature [25].

### Rapamycin treatment

Rapamycin (LC Laboratories, #R-5000, USA) was dissolved in dimethyl sulfoxide (DMSO) and used at a final concentration of 200 nM. This specific dose of rapamycin was chosen based on a previous study showing that it is both effective and non-toxic [26]. Optimization studies were conducted to assess whether rapamycin could inhibit the mTOR signaling pathway. In these studies, rapamycin was added to cells in the IVD group on D6 and D7 in separate experiments. The expression of the phospho-p70 S6 kinase protein was evaluated using western blot analysis (Supplementary Fig. S2).

### Western blotting

On D8, protein extracts from the IVD group were mixed with RIPA buffer containing a protease inhibitor cocktail (Thermo, #89900, USA). Protein concentrations were determined using the BCA assay kit, and 30 µg of protein was loaded per lane onto 10% acrylamide SDS-PAGE gels (Bio-Rad). Protein transfer membranes were blocked with 5% non-fat dry milk in Tris-buffered saline with 1% Tween-20 and incubated overnight at + 4 °C with primary antibodies, including phospho-p70 S6 kinase (Cell Signaling #9234, 1:1000), p70 S6 kinase (Cell Signaling, #2708, 1:1000), and GAPDH (Cell Signaling, #2118, 1:1000). After washing with TBS-T, membranes were incubated with secondary antibodies (horseradish peroxidase-linked anti-rabbit IgG, Vector, #PI-1000–1, USA) at a 1:5000 dilution for 2 h. Chemiluminescent detection was performed using SuperSignal West Pico-Thermo, and western blot bands were visualized with the AZURE C280 MP Imaging System. Density analysis of p-p70S6K/p70S6K in 24 h Rap ( −), 48 h Rap ( −), 24 h Rap ( +), and 48 h Rap ( +) groups was conducted using ImageJ software.

### Immunofluorescence staining of the decidual cells

Cells were collected on D8 of culture from the C, IVD, HU-IVD, C + Rap, IVD + Rap, and HU-IVD + Rap groups. Immunofluorescence staining was performed as described previously [27, 28]. Briefly, after blocking, cells were incubated overnight at 4 °C with primary antibodies against FOXO1 (Rabbit, 1:100, Cell Signaling, #2880, USA), DIO2 (Goat, 1:100, Invitrogen, PA5-18659, USA), and β-galactosidase (Rabbit, 1:400, Cell Signaling, #27198, USA) diluted in 5% BSA-PBS. Rabbit IgG and goat IgG served as the isotype control for appropriate primary antibodies (5 mg/ml, #I-1000–5; 5 mg/ml, #I-5000–5, Vector, USA). Cells were incubated with Alexa Fluor 488 conjugated anti-rabbit and anti-goat secondary antibodies (1:1000, Invitrogen, #A-11008, Abcam, #ab150141, USA) for 1 h at room temperature. Nuclei were stained with Hoechst (1:2000, Invitrogen, #H3570, USA) for 5 min. Cells were visualized under an Olympus BX-61 upright immunofluorescence microscope.

### Identification of the decidual cell population by flow cytometry

On D8, cells from the IVD, HU-IVD, IVD + Rap, and HU-IVD + Rap groups were collected, and the decidual cell subpopulation was identified using FOXO1 (1:200, Cell Signaling, #2880, USA) and DIO2 (1:200, Invitrogen, PA5-18659, Invitrogen, USA) protein expression via flow cytometry. Decidual cells were detached, incubated with primary antibodies FOXO1 and DIO2, washed, and then incubated with secondary antibodies (Alexa Fluor 488 conjugated anti-rabbit and anti-goat secondary antibodies, 1:1000, Invitrogen, #A-11008, Abcam, #ab150141, USA). Senescent cells were identified using the CellEvent™ Senescence Green Flow Cytometry Assay Kit (Invitrogen, #C10840, USA) [25]. The flow cytometry analysis was performed, and an unstained control was used to identify background fluorescence. The unstained control is used as a reference to identify the level of background fluorescence in flow cytometry [29]. Samples were analyzed using CytoFLEX Acquisition and Analysis Software at 488 nm excitation and 525/40 BP fluorescence channels. The percentage of FOXO1 + , DIO2 + , and β-GAL + cells among all cells within the decidual cell gate was determined by flow cytometry, and the data were analyzed using FCS Express Flow Cytometry Software. The percentage of positive cells was analyzed by calculating the proportion of positive events relative to the total number of events $\left[\text{Cell rate}= \left(\frac{\text{Total number of events}}{\text{Number of positive events}}\right)\times 100\right]$ [30].

### Spheroid formation from the human extravillous trophoblast cell line (AC-1M88) and spheroid expansion assay

The hybridoma cell line AC-1M88, derived from primary extravillous trophoblast cells fused with a selectable mutant of JEG-3 choriocarcinoma cells, was obtained from the Deutsche Sammlung von Mikroorganismen und Zellkulturen (DSMZ) (ACC 457) and cultured following the supplier’s instructions. For spheroid formation, 3000 cells were seeded per well in a non-adherent 96-well plate and incubated. Spheroids formed on the second day were collected and added to different cell culture groups on D8. Each group was seeded into a 24-well chamber slide, with one spheroid per well [31, 32]. Decidualization was induced with 8-Br-cAMP at 0.25 mM, as higher concentrations are toxic to AC-1M88 cells [31, 33]. HU was removed from the medium 24 h before adding spheroids to the HU-IVD and HU-IVD + Rap groups. The relative expansion and percent fragmentation of each spheroid were then calculated by dividing the rate in D2 by the rate in D0 [31, 32, 34]. This allowed the spheroid expansion and fragmentation to be measured over time in each experimental group. Spheroids were monitored daily using phase contrast optics, and images were captured using an Olympus IX71 (Zeiss, Germany) inverted microscope. The experiments were independently repeated three times (three technical replicates) in three independent biological samples (*n* = 3). All spheroids that did not meet the predefined criteria in terms of size and shape were excluded from the analysis to preserve the quality of the data [31, 33]. The experiments were carried out in an observer-blinded fashion to minimize the potential for observational bias.

### Establishment of normal pregnancy groups in mice

Six to 8-week-old female BALB/c mice (six animals per group were used) were obtained from Akdeniz University, Turkey, and approved by the Animal Care and Use Committee (ethical approval number 1145/2020.06.007). The sample size was determined based on a power analysis to ensure the statistical robustness and validity of our results. To estimate the minimum number of animals required per group, we followed the guidelines for animal research and power analysis, which recommend consideration of effect size, standard deviation, and the desired power of the study [35]. Animal experiments were carried out in accordance with ARRIVE [36]. Mice were housed in a 12-h light–dark cycle with ad libitum food. Pregnancy groups were established by placing two females with one mature male overnight. The presence of the vaginal plug the following morning was designated as D1 of pregnancy [37–39]. One of the uterine horns was flushed with saline to check for the presence of blastocysts on D4. One hundred microliters of 1% Chicago blue in saline was intravenously injected into the mice to visualize the implantation sites as blue bands on D5 and D6 of pregnancy [40]. The mice were euthanized by cervical dislocation after receiving anesthesia with a mixture of ketamine and xylazine (0.1 ml/10 g body weight, intraperitoneal injection). The uterine samples from each day of pregnancy were collected in the morning (09:00 am) and immediately placed in a fixative solution for immunofluorescence staining and subsequent analysis using microscopy.

### Immunofluorescence staining of paraffin sections

Uterine tissues from mice on different pregnancy days (D4, D5, and D6) were fixed, paraffin-embedded, and sectioned into 5-μm slices on SuperFrost Plus slides. Following deparaffinization and antigen retrieval, sections were permeabilized, blocked, and incubated overnight with primary antibodies (FOXO1, 1:100, Cell Signaling, #2880, USA), (DIO2, 1:100, Invitrogen, PA5-18,659, Invitrogen, USA). After washing, Alexa Fluor 488 conjugated anti-rabbit and anti-goat secondary antibodies (1:1000, Invitrogen, #A-11008, Abcam, #ab150141, USA) were added, and nuclei were stained with DAPI (1:2000, Thermo, #D1306, USA). Sections were coverslipped and observed using an Olympus BX-61 immunofluorescence microscope.

### Statistical analysis

Experimental data were analyzed using descriptive statistics, including the calculation of means and the standard error of the mean. Normality tests were performed using the Shapiro–Wilk test. Parametric data were analyzed using one-way ANOVA test, followed by analysis using the Sidak or Tukey post hoc tests. Non-parametric data were analyzed using the Kruskal–Wallis test, followed by the post hoc Dunn test. Data are presented as mean ± SEM. The specific statistical tests used for each data set are indicated in the figure legends. GraphPad Prism software version 9 was used for all statistical analyses, and statistical significance was set at *p*-value < 0.05.

## Results

Our study found that rapamycin, an mTOR inhibitor and senomorphic agent, effectively reduced markers of senescence without affecting FOXO1 in physiological and induced senescence models. In induced senescence models, rapamycin decreased fragmentation in extravillous trophoblast cells without affecting spheroid expansion. By investigating decidual cell subpopulations during early pregnancy (D4, D5, and D6 in mice), we suggested that the FOXO1-DIO2 signaling axis may initiate decidual senescence in mice during the early stages of pregnancy and implantation.

### Confirmation of the establishment of the IVD and HU-IVD groups

**Fig. 2:**
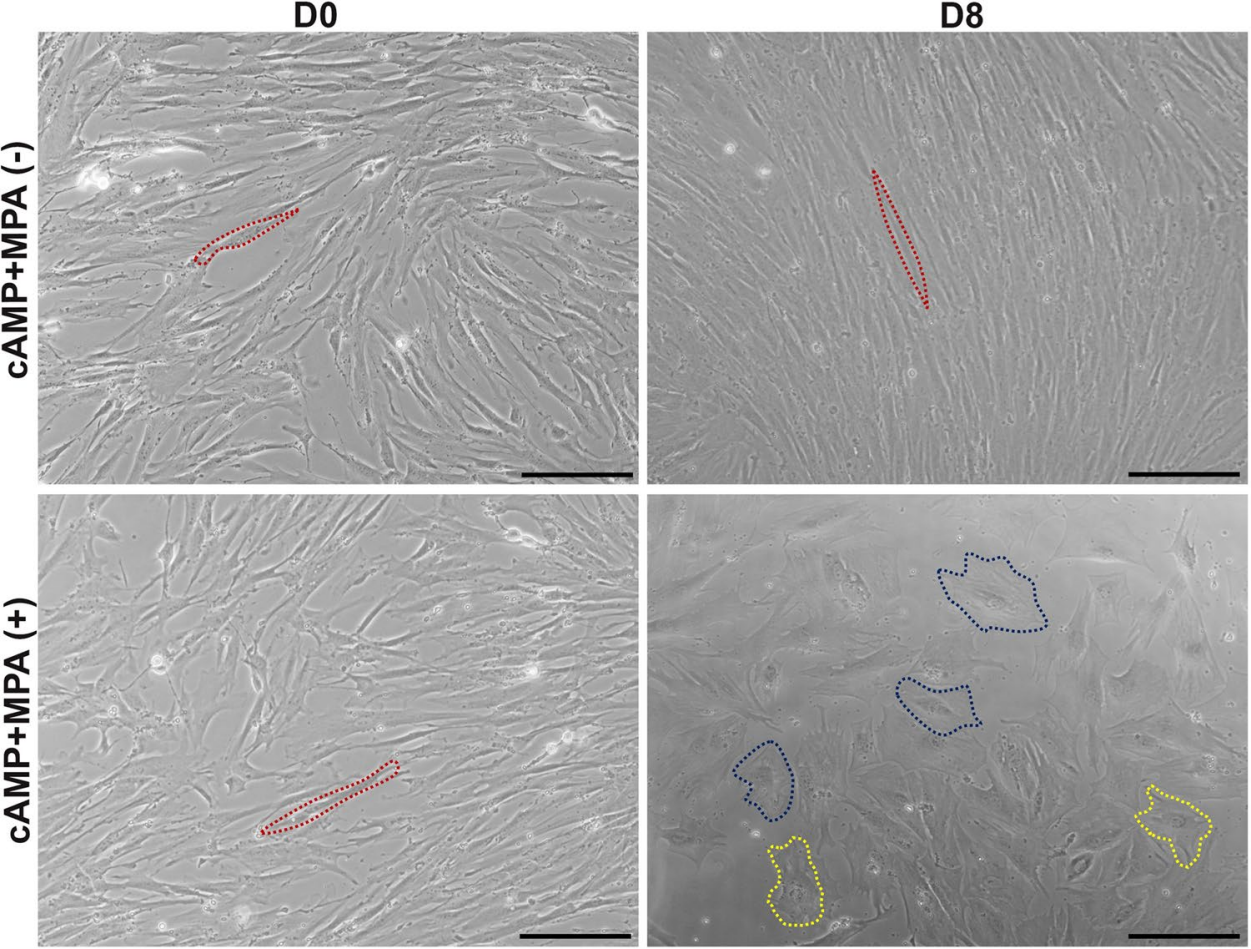
In vitro decidualization. Human endometrial stromal cells (HESCs) (red dash line) undergo a morphological transition from fibroblast-like to epithelial-like appearance during induced decidualization (with cAMP and MPA administration) at 8 days. Blue dashed lines indicate cellular polyploidy, a marker of maturation for decidual cells. In cells undergoing cellular senescence, the nuclei also enlarge with an increase in cellular size [41]. Cells with enlarged nuclei compared to other cells are indicated by the yellow dashed line. The scale bars represent 200 µm. The experiments were independently repeated three times (three technical replicates) in three independent biological samples (*n* = 3)

We evaluated morphology changes in the decidualized group on D8 and observed a transition from fibroblast-like cells to epithelial-like cells, as well as the presence of mature decidual cells characterized by polyploidy, as shown in Fig. 2. The enlarged nuclei were observed in cells undergoing cellular senescence. The endometrial stromal cells in the control group maintained their fibroblast-like morphology throughout the 8 days of culture. 0.1 mM HU treatment caused a statistically significant increase in the percentage of senescence associated-β-galactosidase (SA-β-Gal)–positive cells as assessed by CellEvent™ Senescence Green Staining compared to 0.2 mM HU and 0.5 mM HU treatments (*p* = 0.001). However, there was no statistically significant difference between the 0.2 and 0.5 mM HU treatments (*p* > 0.05). Furthermore, cells were suspended in a monolayer culture, indicating a toxic effect on the cells when treated with 0.2 mM and 0.5 mM HU. Therefore, 0.1 mM HU was determined to be the optimal dose for generating the HU-IVD model (Supplementary Fig. S1).

### Confirmation of the inhibition of the mTOR signaling pathway in the IVD group

The level of p-p70S6K protein expression as a marker of mTOR signaling was evaluated by western blot analysis. The results showed a statistically significant decrease in p-p70S6K expression in both the 24 h Rap ( +) and 48 h Rap ( +) groups when compared to the 24 h Rap ( −) and 48 h Rap ( −) groups (*p* < 0.001). Furthermore, there was a statistically significant decrease in p-p70S6K expression in the 48 h Rap ( +) group when compared to the 24 h Rap ( +) group (*p* = 0.006), indicating that rapamycin effectively inhibited mTOR signaling when applied for 48 h. Based on these findings, the C + Rap, IVD + Rap, and HU-IVD + Rap treatment groups were formed by adding 200 nM rapamycin to the control, physiological decidualization, and induced senescence groups on D6 (Supplementary Fig. S2).

### Rapamycin treatment reduces the number of senescent cells in the presence of HU-IVD

**Fig. 3:**
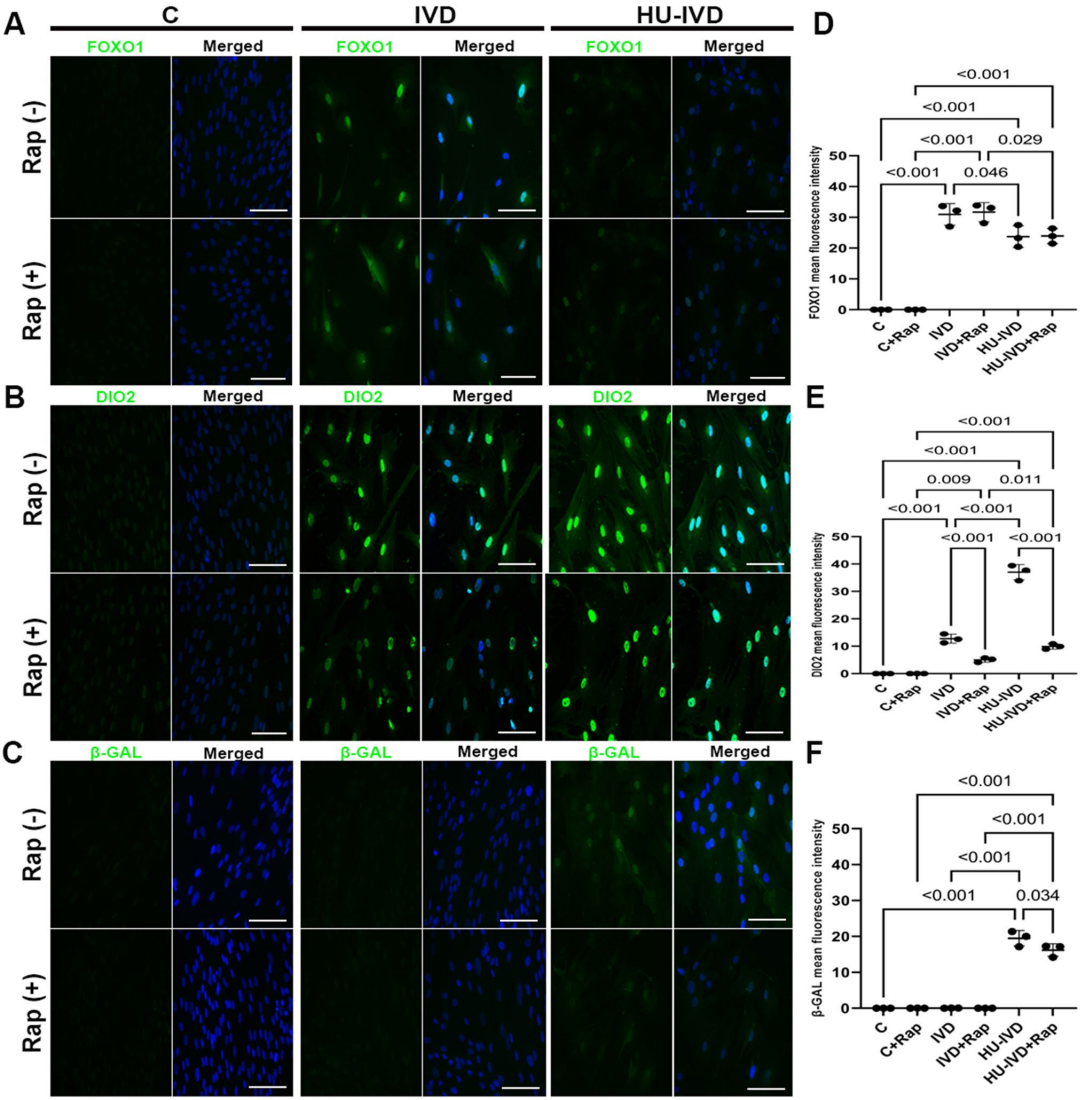
Immunofluorescence staining. On D8 of the culture, cells were collected from the C, IVD, HU-IVD, C + Rap, IVD + Rap, and HU-IVD + Rap groups, and immunofluorescence staining was performed. Images represent immunofluorescence staining of cells with **A** FOXO1 (Alexa Fluor 488-green), **B** DIO2 (Alexa Fluor 488-green), and **C** β-GAL (Alexa Fluor 488-green) and counterstaining of nuclei with Hoechst dye (blue). The scale bars represent 200 µm. **D** Mean fluorescence intensity values of FOXO1, **E** DIO2, and **F** β-GAL in C, IVD, HU-IVD, C + Rap, IVD + Rap, and HU-IVD + Rap groups. Data are presented as mean ± SEM. Statistically significant differences (*p* < 0.05) between the selected groups (C and IVD, C and HU-IVD, IVD and HU-IVD, C + Rap and IVD + Rap, C + Rap and HU-IVD + Rap, IVD + Rap and HU-IVD + Rap, C and C + Rap, IVD and IVD + Rap, and HU-IVD and HU-IVD + Rap) were shown using one-way ANOVA followed by post hoc Sidak multiple comparison test. *p*-values < 0.05 are appended to the graphs. The experiments were independently repeated three times (three technical replicates) in three independent biological samples (*n* = 3)

On D8 of culture, decidual cell subpopulations were analyzed in all groups with and without rapamycin through immunofluorescence staining for FOXO1 (Fig. 3A), DIO2 (Fig. 3B), and β-GAL (Fig. 3C).

The results showed that the expression of FOXO1, which is a key transcription factor for decidualization, was significantly increased in both the IVD and HU-IVD groups when compared to the control group (*p* < 0.001). Moreover, the HU-IVD group showed a statistically lower expression of FOXO1 compared to the IVD group (*p* = 0.046). Furthermore, there was a significant increase in FOXO1 expression in the IVD + Rap and HU-IVD + Rap groups compared to the C + Rap group (*p* < 0.001). The HU-IVD + Rap group had lower FOXO1 expression than the IVD + Rap group (*p* = 0.029). However, there was no significant difference in FOXO1 expression between the C versus C + Rap, HU-IVD versus HU-IVD + Rap, and IVD versus IVD + Rap groups (*p* > 0.05) (Fig. 3D).

The expression of DIO2 significantly increased in the IVD group and in the HU-IVD group compared to the control group (*p* < 0.001). In addition, the expression of DIO2 was significantly higher in the HU-IVD group than in the IVD group (*p* < 0.001). In the rapamycin treatment groups, DIO2 expression was significantly higher in the IVD + Rap and HU-IVD + Rap groups than in the C + Rap group (*p* = 0.009 and *p* < 0.001, respectively). In addition, the expression of DIO2 was significantly higher in the HU-IVD + Rap group compared to the IVD + Rap group (*p* = 0.011). However, DIO2 expression was significantly lower in the IVD + Rap group than in the IVD group and in the HU-IVD + Rap group than in the HU-IVD group (*p* < 0.001) (Fig. 3E).

The expression of β-GAL was found to be significantly increased in the HU-IVD group compared to the control and IVD groups (*p* < 0.001). However, there was no statistically significant difference in β-GAL expression between the C and IVD groups (*p* > 0.05). The HU-IVD + Rap group showed significantly higher β-GAL expression compared to the C + Rap and IVD + Rap groups (*p* < 0.001). There was no significant difference in β-GAL expression between the C + Rap and IVD + Rap groups (*p* > 0.05). After rapamycin treatment, β-GAL expression decreased significantly in the HU-IVD + Rap group compared to the HU-IVD group (*p* = 0.034) (Fig. 3F).

**Fig. 4:**
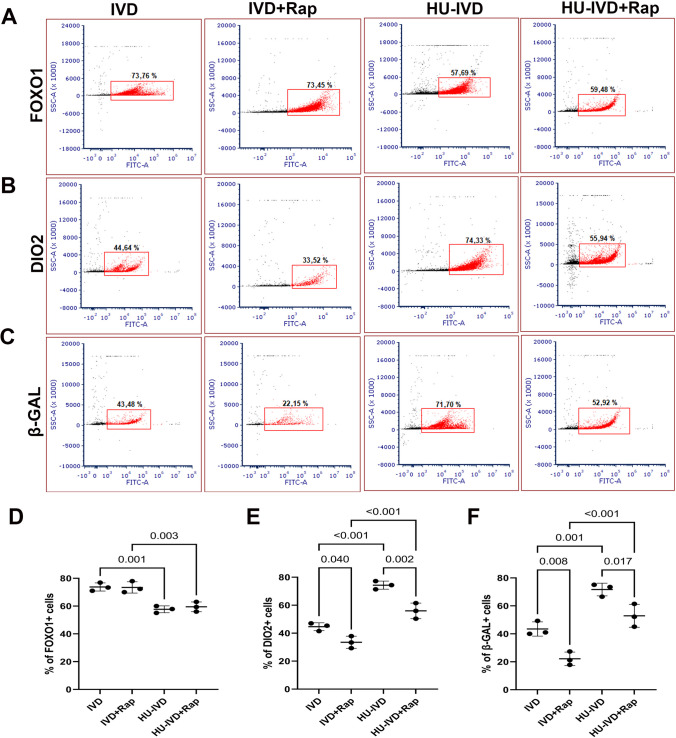
Identification of the decidual cell subpopulation by flow cytometry. Cells were analyzed for forward light scattering (FSC) and side light scattering (SSC) characteristics. Specific cell populations were then identified by antibody-based staining. Representative dot plot of flow cytometry data showing identification strategy for** A** FOXO1 + cell populations,** B** DIO2 + cell populations,** C** β-GAL + cell populations, **D** percentage of FOXO1 + cell populations,** E** DIO2 + cell populations, and **F** β-GAL + cell populations. Data are presented as mean ± SEM. Statistically significant differences (*p* < 0.05) between the selected groups (C and IVD, C and HU-IVD, IVD and HU-IVD, C + Rap and IVD + Rap, C + Rap and HU-IVD + Rap, IVD + Rap and HU-IVD + Rap, C and C + Rap, IVD and IVD + Rap, and HU-IVD and HU-IVD + Rap) were shown using one-way ANOVA followed by post hoc Sidak multiple comparison test. *p*-values < 0.05 are appended to the graphs. The experiments were independently repeated three times (three technical replicates) in three independent biological samples (*n* = 3)

By using flow cytometry, the expression of FOXO1 (Fig. 4A), DIO2 (Fig. 4B), and β-GAL (Fig. 4C) was analyzed in the IVD, HU-IVD, IVD + Rap, and HU-IVD + Rap groups on D8 of the culture. This was done to identify the decidual cell subpopulations that emerged during the senescence process in both IVD and HU-IVD models and to correlate the findings with immunofluorescence results. The percentage of cells expressing FOXO1 was significantly higher in the IVD group compared to the HU-IVD group (*p* = 0.001). Similarly, the percentage of FOXO1-positive cells was significantly higher in the IVD + Rap group compared to the HU-IVD + Rap group *(p* = 0.003). However, there was no statistically significant difference in the percentage of FOXO1-positive cells between the IVD and IVD + Rap groups, as well as between the HU-IVD and HU-IVD + Rap groups (*p* > 0.05) (Fig. 4D).

The percentage of DIO2-positive cells was significantly greater in the HU-IVD group compared to the IVD group (*p* < 0.001). Similarly, the percentage of DIO2-positive cells was significantly higher in the HU-IVD + Rap group compared to the IVD + Rap group (*p* < 0.001). Moreover, following treatment with rapamycin, there was a significant reduction in the percentage of DIO2-positive cells in both the IVD + Rap group compared to the IVD group and the HU-IVD + Rap group compared to the HU-IVD group (*p* = 0.040 and* p* = 0.002, respectively) (Fig. 4E).

The percentage of cells positive for β-GAL was found to be significantly higher in the HU-IVD group than in the IVD group (*p* = 0.001). Similarly, the percentage of β-GAL + cells was significantly higher in the HU-IVD + Rap group compared to the IVD + Rap group (*p* < 0.001). After rapamycin treatment, there was a significant decrease in the percentage of β-GAL + cells in both the IVD + Rap and HU-IVD + Rap groups when compared to their respective control groups (*p* = 0.008 and *p* = 0.017, respectively) (Fig. 4F).

### Induced senescence leads to poor-quality blastocyst-like fragmentation of spheroids, and treatment with rapamycin reduces fragmentation

**Fig. 5:**
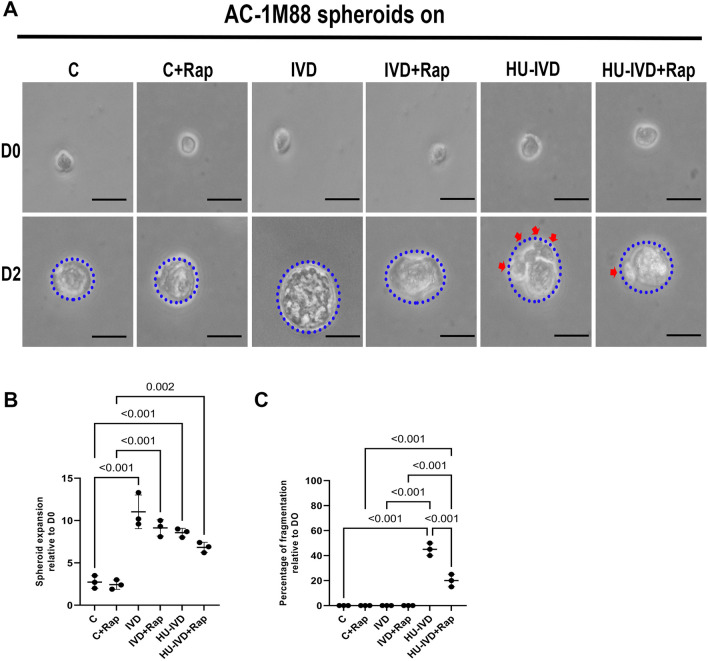
Spheroid formation from AC-1M88 human extravillous trophoblast cells and subsequent spheroid expansion assay. **A** Phase contrast micrographs of spheroids were obtained immediately (D0) and 48 h later (D2). The blue dashed lines represent the measured expansion of each spheroid, while the red arrowhead indicates fragmentation. The scale bars represent 200 µm.** B** The relative expansion of each spheroid was calculated by dividing the spheroid expansion at D2 by the spheroid expansion at D0. The results of the spheroid expansion experiment are expressed as the ratio of the spheroid expansion at D2 to that at D0. **C** The percentage of fragmentation in each spheroid was calculated as the percentage of fragmentation in D2 divided by D0. Data are presented as mean ± SEM. Statistically significant differences (*p* < 0.05) between the groups were demonstrated using a Kruskal–Wallis test followed by post hoc Dunn’s multiple comparison test. *p*-values < 0.05 are appended to the graphs. The experiments were independently repeated three times (three technical replicates) in three independent biological samples (*n* = 3)

AC-1M88 cells were grown for 48 h in the C, IVD, HU-IVD, C + Rap, IVD + Rap, and HU-IVD + Rap groups (Fig. 5A). The results showed a significant increase in spheroid expansion in the IVD and HU-IVD groups compared to the control group (*p* < 0.001). However, although there was a decrease in spheroid expansion in the HU-IVD group compared to the IVD group, this was not statistically significant (*p* > 0.05) (Fig. 5B). In the rapamycin-treated groups, the IVD + Rap group showed a statistically significant increase in spheroid expansion compared to the C + Rap group (*p* < 0.001). Similarly, there was a statistically significant difference in spheroid expansion in the HU-IVD + Rap group compared to the C + Rap group (*p* = 0.002). However, no statistically significant effect on spheroid expansion was observed between the C and C + Rap, IVD and IVD + Rap, and HU-IVD and HU-IVD + Rap groups (*p* > 0.05) (Fig. 5B).

On the other hand, there was a statistically significant increase in fragmentation-like structures, which is the differential diagnosis of blastocysts with poor morphology, in the HU-IVD group compared to the C and IVD groups (*p* < 0.001). Although there was a statistically significant increase in fragmentation-like structures in the HU-IVD + Rap group compared to the C + Rap and IVD + Rap groups (*p* < 0.001), there was a statistically significant decrease in the fragmentation-like structures in the HU-IVD + Rap group compared to the HU-IVD group (*p* < 0.001) (Fig. 5C).

### The FOXO1-DIO2 signaling *axis* can induce decidual senescence in early pregnancy and implantation in mice

**Fig. 6:**
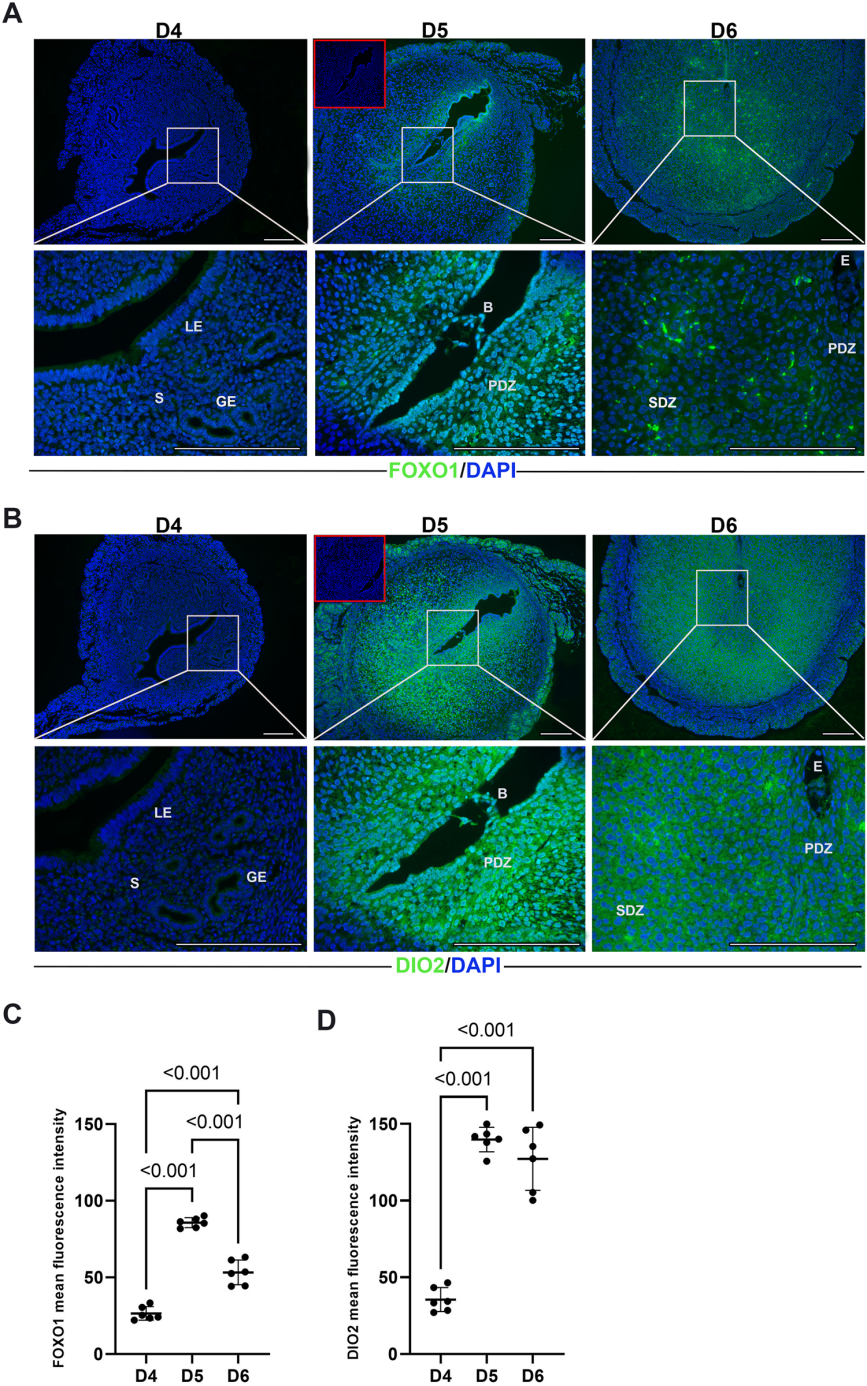
Immunofluorescence staining on uterine specimens collected during pregnancy D4, D5, and D6. Immunofluorescence staining was performed to visualize the localization of **A** FOXO1 (Alexa Fluor 488-green) and **B** DIO2 (Alexa Fluor 488-green) in uterine samples collected on D4 (*n* = 6), D5 (*n* = 6), and D6 (*n* = 6) of pregnancy. DAPI (blue) was used to counterstain the nuclei. The images in the red square represent negative control. The negative control indicates the specificity of FOXO1 and DIO2 immunofluorescence staining. The scale bars represent 200 µm. **C** Mean fluorescence intensity values of FOXO1 and **D** DIO2 on D4, D5, and D6. Data are presented as mean ± SEM. Statistically significant differences (*p* < 0.05) between all groups were indicated using one-way ANOVA followed by post hoc Tukey multiple comparison test. *p*-values < 0.05 are appended to the graphs. The experiments were independently repeated three times (three technical replicates) in six independent biological samples (*n* = 6). LE, luminal epithelium; GE, glandular epithelium; S, stroma; B, blastocyst; E, embryo; PDZ, primary decidual zone; SDZ, secondary decidual zone

We conducted an in vivo analysis of decidual cell subpopulations during the pre-receptive period (D4), receptive period (D5), and refractory period (D6). Immunofluorescence staining was used to examine the expression of FOXO1 (Fig. 6A) and DIO2 (Fig. 6B). The subcellular localization of FOXO1 and DIO2 proteins was different on various days of pregnancy in mice.

FOXO1, which was cytoplasmically localized on the morning of gestation D5, was also in the cytoplasm on the morning of gestation D6 (Fig. 6A). DIO2 was predominantly localized in the cytoplasm of the cells on morning D5 of gestation, whereas it was only in the cytoplasm on morning D6 of gestation (Fig. 6B).

A significant increase in the expression of FOXO1 was observed on D5 and D6 of pregnancy compared to D4 (*p* < 0.001). Furthermore, there was a significant decrease in FOXO1 expression on D6 of pregnancy, particularly in the primary decidual zone (PDZ), compared to D5 (*p* < 0.001). On D6 of pregnancy, the expression of FOXO1 expanded towards the secondary decidual zone (SDZ) (Fig. 6C).

DIO2 expression was significantly upregulated in both PDZ and SDZ on D5 and D6 in comparison with D4 of pregnancy (*p* < 0.001). However, there was no significant difference in DIO2 expression between D5 and D6 (*p* > 0.05). On D6 of pregnancy, there was widespread and high DIO2 expression in SDZ compared to PDZ (Fig. 6D).

## Discussion

Endometrial remodeling during embryo implantation is regulated by the spatiotemporal extent of decidual senescence and the efficiency of immune clearance processes [8]. During decidualization, senescent decidual cells are cleared by immune cells to maintain a healthy environment. In an induced senescence environment, the increased number of senescent decidual cells in the environment causes decidualization defects and creates a long-term pro-inflammatory environment that prevents embryo implantation [42]. In decidual subsets identified using high-throughput single-cell RNA sequencing, the presence of an increased number of senescent decidual cells in the environment has been shown to be associated with excessive decidual senescence and defects in the formation of the placental-decidual interface [7, 8].

To determine the effects of HU on decidualization, mice were injected with HU on D4 of pregnancy and on D5 and D6 of artificial decidualization. Impaired decidualization was accompanied by a decrease in implantation site weights. In the same study, the effect of HU on decidualization in vitro was also evaluated, and after HU treatment of isolated mouse endometrial stromal cells, decreased proliferation activity was observed in decidual cells due to increased decidual senescence [43]. In this study, we chose to use HU to induce the senescence of decidual cells.

It has been reported that knockdown of FOXO1 using siRNA in endometrial stromal cells not only causes a decrease in decidual cell markers but also causes a significant decrease in senescence-associated beta-galactosidase activity, and FOXO1 causes acute senescence of decidual cells in an IL-8-dependent manner [8]. Similarly, it has been reported that FOXO1 knockdown suppresses decidual senescence in endometrial stromal cells [44]. In another study, it was demonstrated that the process of decidual senescence is primarily triggered by upregulation of FOXO1 expression [45]. In our study, increased FOXO1 expression was observed in the physiological decidualization group compared to the control and induced senescence groups, suggesting that FOXO1 may contribute to physiological decidual senescence. Furthermore, rapamycin did not cause any change in FOXO1 expression, suggesting that FOXO1 is not regulated by rapamycin during decidual senescence.

The conversion of the prohormone thyroxine (T4) to the bioactive triiodothyronine (T3) by DIO2 indicates increased energy metabolism in cells and has been observed to be upregulated in senescent decidual cells [7]. In recurrent pregnancy losses, increased DIO2 expression is observed due to pathological decidual senescence [46]. Furthermore, DIO2, expressed as a marker of senescent decidual cells, also regulates numerous genes encoding extracellular matrix remodeling factors associated with senescence [7]. In the physiological model of decidualization, the presence of cells expressing DIO2 supports the idea that decidualization undergoes an acute senescence process. In the HU-treated induced senescence model, DIO2 expression was significantly increased compared to the physiological decidualization model, suggesting an increased presence of senescent cells in induced senescence models. After treatment with rapamycin, we detected a decrease in the expression of this marker in both the IVD + Rap and HU-IVD + Rap groups, suggesting that rapamycin treatment influences senescent cells in the environment.

Our flow cytometry data is generally aligned with immunofluorescence results, except for FOXO1 and β-GAL expressions. While FOXO1 staining exhibited low expression in the HU-IVD and HU-IVD + Rap groups in immunofluorescence, flow cytometry showed a percentage of ~ 57 and ~ 59 positively stained cells, respectively. For β-GAL, no expression was seen in the IVD and IVD + Rap groups in immunofluorescence, but flow cytometry revealed a percentage of ~ 41 and ~ 20 positively stained cells, respectively. We postulated that these differences are due to differences in the sensitivity of the assays and the capacity of the flow cytometry method to respond more sensitively even to low levels of antigen expression [47, 48].

However, it is important to note that β-GAL serves as a marker for senescent cells, and their increased presence indicates the transition from physiological to pathological decidual senescence [7, 49]. This shift may lead to impaired decidualization, increased senescent cells, inadequate support for embryo development, and activation of prostaglandin-mediated signaling, potentially causing spontaneous preterm birth [49]. Our immunofluorescence and flow cytometry results indicate that rapamycin treatment decreased β-GAL expression in in vitro physiological and induced senescence models, suggesting its effectiveness on senescent cells and its potential to alleviate pathological conditions.

A recent study found that senescent decidual cells create a favorable implantation environment for embryo expansion and attachment [46]. However, elevated decidual senescence levels lead to impaired maternal–fetal interaction, slow spheroid expansion, and increased fragmentation [7, 31, 46], affecting healthy embryonic development [50]. In IVF/ICSI, daily monitoring of blastocyst fragmentation reveals poor-quality blastocysts with over 20% fragmentation [51]. Before and after rapamycin treatment, no fragmentation of AC-1M88 spheroids was observed in control or physiological decidualization groups, whereas AC-1M88 spheroids cultured in induced senescence environments showed fragmentation reflecting the morphology of poor-quality blastocysts (~ 40%). However, after rapamycin treatment, the fragmentation of AC-1M88 spheroids cultured in an induced senescence environment was statistically reduced (~ 20%). This effect suggests that rapamycin treatment may positively affect the crosstalk between the embryo and the intrauterine environment by alleviating the pathological senescence environment, thereby potentially increasing implantation capacity.

FoxO1 expression changes spatiotemporally during implantation in the peri-implantation period in mice [40]. Increased FOXO1 expression in the PDZ on the morning of D5 of pregnancy was followed by decreased FOXO1 expression in the PDZ, which shifted to the SDZ on the morning of D6 of pregnancy. However, DIO2 expression was also significantly higher in the newly formed SDZ at D5 of pregnancy, whereas it decreased in the PDZ and shifted highly to the SDZ at D6 of pregnancy. The increased expression of DIO2 and FOXO1 in the PDZ at D5 of gestation may indicate their possible joint contribution to decidualization. Protein transport in cells is a complex process regulated by multiple factors [52]. Activation of the FOXO1 protein occurs in the nucleus of the cell [53]. In our study, FOXO1, which was found in its cytoplasmic localization on the morning of D5 of pregnancy when endometrial receptivity occurs in mice, was also in the cytoplasm on the morning of D6 of pregnancy when the refractory phase was observed.

DIO2 exhibits a predominantly cytoplasmic expression pattern on morning D5 of gestation. However, by the morning of D6 of pregnancy, its expression is exclusively localized to the cytoplasm. Activation of the DIO2 protein occurs in the cytoplasm of the cell [54]. In mice, DIO2 protein was found to be more active in the cytoplasm on the morning of D6 of pregnancy, when implantation continues but the endometrium enters the refractory phase, rather than on the morning of D5 of pregnancy, when endometrial receptivity is acquired, suggesting the presence of decidual senescence in mice under physiological conditions, especially on D6 of pregnancy. Furthermore, FOXO1 expression at D5 of gestation was followed by DIO2 expression in an active form in the cytoplasm at D6 of gestation. Taken together, our results suggest that the FOXO1-DIO2 signaling axis may be involved in the physiological decidual senescence process during early pregnancy and implantation days in mice. It has also been shown in the literature that DIO2 is a direct transcriptional target of FOXO1 and that the FOXO1-DIO2 signaling axis governs cellular processes in vitro and in vivo in cardiomyocytes [55]. This study has also underscored the role of the FOXO1-DIO2 signaling axis in eliciting cellular stress within cardiomyocytes. Analogously, the process of embryo implantation serves as a significant source of cellular stress for both the developing embryo—manifested through mechanical, hypoxic, and oxidative stress—and the maternal tissues, which experience an inflammatory response, tissue remodeling, and a surge in hormonal activity. Therefore, we speculate that the FOXO1-DIO2 signaling axis drives physiological decidual senescence in pregnant mice.

However, it should be kept in mind that the cellular localization of proteins can be complex and dynamic, and proteins may have more than one location within the cell depending on their specific role [56]. Therefore, further research is needed to fully understand the mechanisms underlying the transport and perhaps the signaling axis of FOXO1 and DIO2 proteins involved in decidual senescence and their role in the regulation of decidual senescence during early pregnancy in mice.

**Fig. 7:**
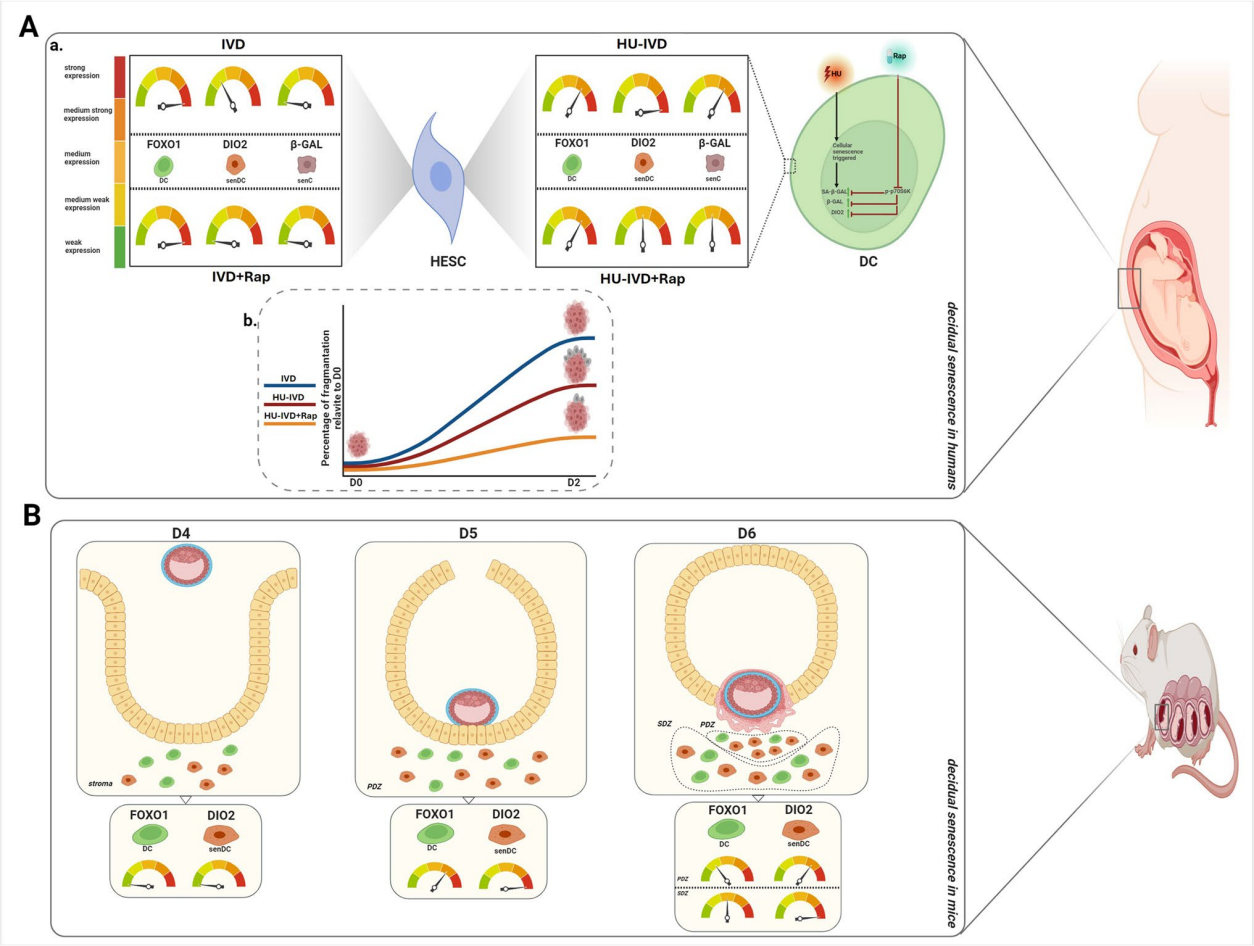
Schematic summary. **A** Decidual senescence in humans. (a) Hydroxyurea (HU) application to decidual cells induced cellular senescence and increased SA-β-Gal, β-GAL, and DIO2 expression. After rapamycin treatment, p-p70S6K protein expression and cellular senescence markers decreased. Using human endometrial stromal cells (HESC): physiological decidualization (in vitro decidualization-IVD), rapamycin-treated physiological decidualization (IVD + Rap), HU-treated induced senescence (HU-IVD), and rapamycin-treated HU-IVD (HU-IVD + Rap). In the IVD group, FOXO1 was strongly expressed, whereas DIO2 was moderately weak, and β-GAL was weakly expressed. After rapamycin treatment in the IVD group, FOXO1 expression remained unchanged (strong), while DIO2 and β-GAL decreased and were weakly expressed. In the HU-IVD group, FOXO1 was medium strong, DIO2 was strong, and β-GAL was medium strong. After rapamycin treatment in the HU-IVD group, FOXO1 expression remained unchanged (medium strong), while DIO2 and β-GAL decreased and were medium expressed. Rapamycin treatment decreased senescence markers and regulated decidual senescence without affecting decidualization. (b) AC-1M88 spheroids, an extravillous trophoblast cell line mimicking blastocyst, were co-cultured with IVD, HU-IVD, and HU-IVD + Rap groups for 2 days. In the physiological decidualization group, no fragmentation was observed in AC-1M88 spheroids, whereas spheroids in the induced senescence group exhibited poor-quality blastocyst-like fragmentation. After rapamycin treatment, the number of fragmentations in the HU-IVD group decreased. **B** Decidual senescence in mice. Physiological decidual senescence in mice proceeds differently in the mornings of D4, D5, and D6 of pregnancy. On the fourth day of pregnancy, when the endometrium is in a pre-receptive state, FOXO1 and DIO2 are expressed weakly. On the fifth day of pregnancy, when the endometrium is in a receptive state and the blastocyst is in contact with the endometrial epithelium, FOXO1 is medium strong, and DIO2 is strong in the primary decidual zone (PDZ). On the sixth day of pregnancy, when blastocyst invasion occurs and the endometrium becomes refractory, the expression of markers in the primary and secondary decidual zones (SDZ) differs. On the sixth day of pregnancy, FOXO1 is medium weak, and DIO2 is medium strong in the PDZ. However, in the SDZ, where mature decidual cells are observed, FOXO1 is medium expressed, and DIO2 is strongly expressed

In conclusion, our results highlight the importance of rapamycin administration on decidual cell subpopulations and endometrial tissue function in physiological and induced senescence processes. Moreover, focusing on key regulators of physiological and induced senescence processes, namely, FOXO1-DIO2 signaling pathways, contributes to our understanding of these important molecular mechanisms and reveals the effect of rapamycin treatment. This study increases our knowledge of decidual senescence in humans and mice, albeit limited by its dependence on in vitro models and partial understanding of rapamycin treatment. It also contributes to the development of new therapeutic protocols for regulating induced senescence and offers novel approaches and ideas for the treatment of pregnancy-related pathologies in clinical settings. The main conclusions are summarized in Fig. 7.

## Supplementary Information

Below is the link to the electronic supplementary material.

Supplementary file1 (JPG 544 KB)

Supplementary file2 (JPG 3194 KB)

Supplementary file3 (DOCX 15 KB)

## Data Availability

Data supporting the findings of this study are available upon reasonable request from the corresponding author.
