# Supplementary figures and images for: Deciphering the role of rapamycin in modulating decidual senescence: implications for decidual remodeling and implantation failure
Source: J Assist Reprod Genet. 2024 Jul 27;41(9):2441–56. doi: 10.1007/s10815-024-03207-5 (PMC11405573; doi:10.1007/s10815-024-03207-5)

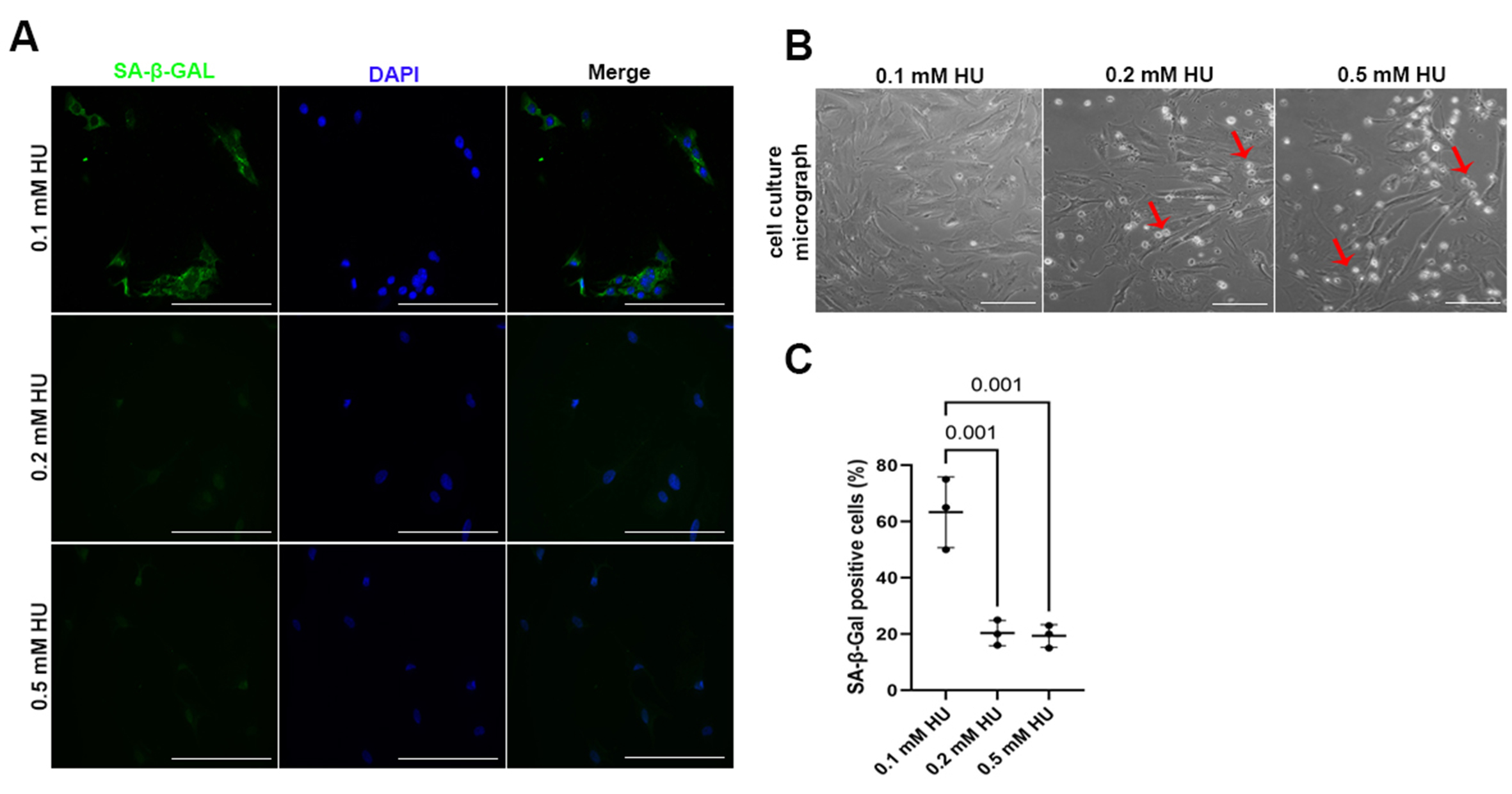

Supplement: Supplementary file 1 — Supplementary file1 (JPG 544 KB) [file 10815_2024_3207_MOESM1_ESM.jpg]

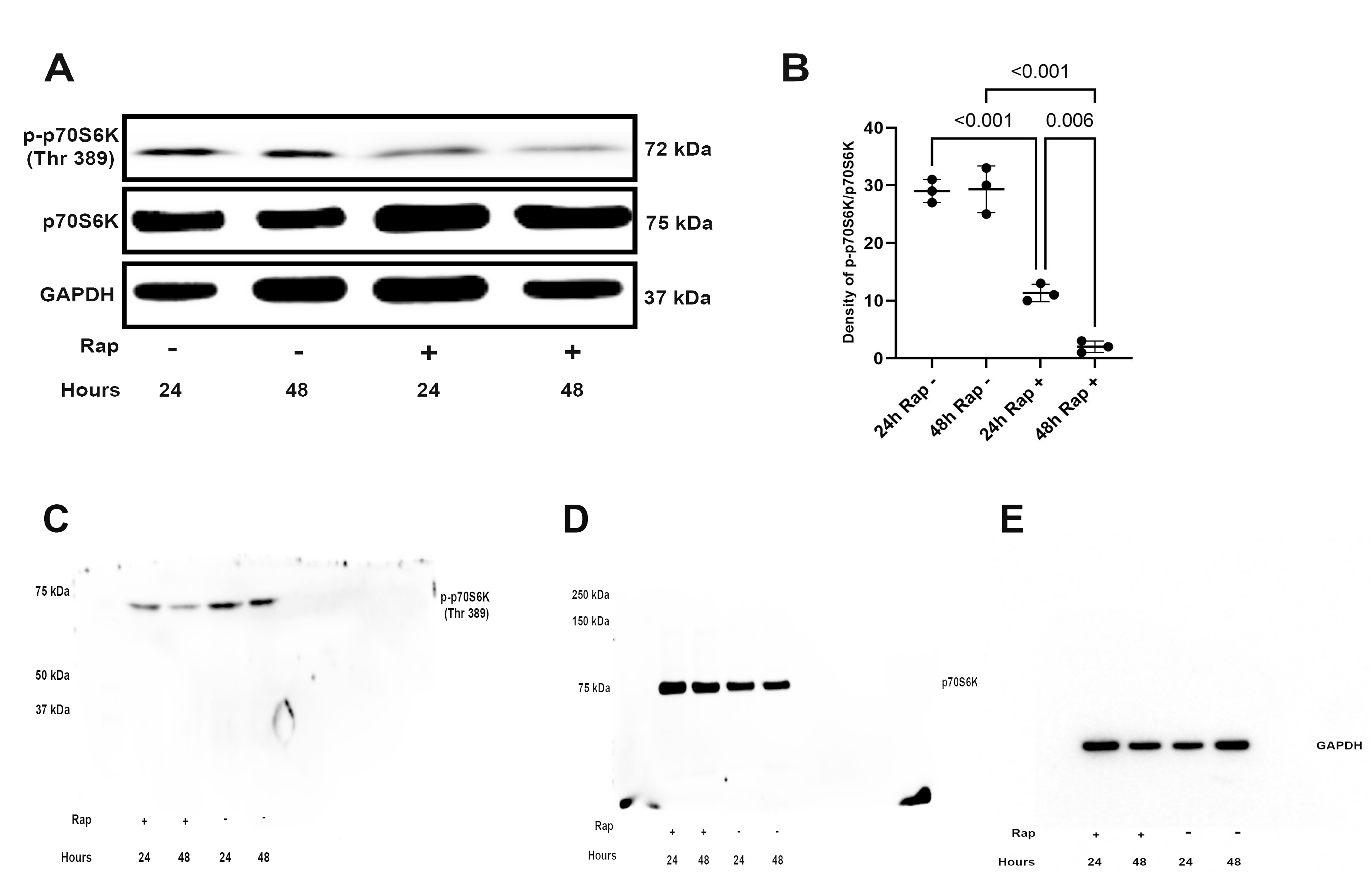

Supplement: Supplementary file 2 — Supplementary file2 (JPG 3194 KB) [file 10815_2024_3207_MOESM2_ESM.jpg]
