# Supplementary material for: Deciphering the role of rapamycin in modulating decidual senescence: implications for decidual remodeling and implantation failure
Source: J Assist Reprod Genet. 2024 Jul 27;41(9):2441–56. doi: 10.1007/s10815-024-03207-5 (PMC11405573; doi:10.1007/s10815-024-03207-5)
Supplement: Supplementary file 3 — Supplementary file3 (DOCX 15 KB) [file 10815_2024_3207_MOESM3_ESM.docx]

**Supplementary data**

**Supplementary Figure S1 Induction of cellular senescence by HU for HU-IVD group.** **(A)** The induced senescence group was performed at three different concentrations (0.1 mM, 0.2 mM, and 0.5 mM HU) for optimization experiments. An increase in the expression of SA-β-GAL a hallmark of senescence onset, was visualized using an Olympos BX-61 fluorescence microscope and could be detected with the CellEvent™ Senescence Green Staining. Senescent cells were identified by green staining. **(B)** Concentrations above 0.1 mM (especially 0.2 mM and 0.5 mM) led to cell toxicity, causing monolayer cells to become suspended cells (red arrow). Morphologic cell changes were monitored with phase contrast optics and images were captured using an Olympos IX71 (Zeiss, Germany) inverted microscope. The scale bar for all images is 200 µm. **(C)** The percentage of SA-β-GAL+ cells was calculated. Data are presented as mean ± SEM. Statistically significant differences (*p*<0.05) between all groups were indicated using one-way ANOVA followed by post hoc Tukey multiple comparison test. *P*-values < 0.05 are appended to the graphs. Experiments were independently repeated three times (3 technical replicates) in three independent biological samples (n=3).

**Supplementary Figure S2 The initiation of rapamycin treatment and assessment of its efficacy. (A)** Representative western blots of the p-p70S6K, and p70S6K in the 24h Rap (-), 48h Rap (-), 24h Rap (+), and 48h Rap (+) groups. GAPDH served as a loading control. **(B)** Density of p-p70S6K/p70S6K in the 24h Rap (-), 48h Rap (-), 24h Rap (+), and 48h Rap (+) groups. Data are presented as mean ± SEM. Statistically significant differences (p<0.05) between the selected groups [(24h Rap (-) & 48h Rap (-), 24h Rap (+) & 48h Rap (+), 24h Rap (-) & 24h Rap (+), and 48h Rap (-) & 48h Rap (+)] were shown using one-way ANOVA followed by post hoc Sidak multiple comparison test. Statistically significant differences (*p*<0.05) between the groups were demonstrated using a one-way ANOVA test followed by post hoc Sidak multiple comparison test. *P*-values < 0.05 are appended to the graphs. **(C)** p-p70S6K (Thr-389), **(D)** p70S6K, **(E)** GAPDH represent complete membrane images in western blot analysis. Experiments were independently repeated three times (3 technical replicates) in three independent biological samples (n=3).
